# Teaching Anatomy in Medical Schools

**Published:** 1893-10

**Authors:** B. T. Flavin

**Affiliations:** Demonstrator of Anatomy, Medical Department, University of Texas, Galveston


For Texas Medical Journal.

### TEACHING ANATOMY IN MEDICAL SCHOOLS.

BY B. T. FLAVIN, M. D.,
(Demonstrator of Anatomy, Medical Department, University of Texas.)

[Read before Galveston County Medical Society, May, 1893.]

"A GOOD anatomist is a poor surgeon, or at least a very timid one." What student or graduate in medicine has not heard this oft-repeated saying, this weighty wisdom-laden dictum, this thrice-welcome solace of sluggard students, this *refugium asinorum medicarum*, this silly superficial apology of superficial and incompetent teachers of anatomy? "Too much learning will bring a man to the gallows" was the sapient remark with which a schoolmate of mine in the days of my boyhood persuaded his "gran'daddy" to withdraw him from school after he had learned what were then known as "vulgar fractions" and "double rule of three." I do not know whether the remark which won my schoolmate his freedom was original with him, but I do know that the saying about the good anatomist being a poor surgeon has been very generally attributed to Billroth of Vienna—by way, I suppose, of making it respectable. Whether the saying be of respectable origin or not, there is no denying the fact that in many medical schools of the South and West it is highly and in all but universally respected. Both through students and graduates from those schools and through reports direct and indirect of one another, the fact has been well ascertained that students in these schools receive such a training in anatomical work as should make them, on the reputed authority of Billroth, not geniuses, but veritable genii of the surgeon's saw and scalpel.

To advance an argument against this chosen motto of the citadels of small knowledge would be an insult to the intelligence of any doctor of medicine who did not purchase his diploma at a job printing office, or who did not get it from a school where the good surgeons are the poor anatomists, and vice versa; yet, for the benefit of young aspirants whose untrained minds are as readily impressed by plausible falsehood as by wholesome truth, I would point out that if there be such a thing as a good surgeon whose knowledge of anatomy is very limited, that surgeon would surely be a better one if his knowledge of anatomy were more certain and extensive. As to the timidity which might result from sound anatomical knowledge, it is comparable to the timidity which differentiates the sea-captain who knows the exact location of the rocks and shoals that lie in his course from the heroic sea-dog who knows not and recks not whetner the bed of the sea on which he is sailing is twenty feet or twenty fathoms down. Let the young student ask himself with which captain he would prefer to risk his own life; or again, other things being equal, into which surgeon's hands he would prefer to fall in case of need, the surgeon who knew anatomy or the surgeon who didn't. The truth is, it is not knowledge that is timid, but ignorance that is audacious. "The fool will rush where the angel fears to tread."

"You may know Gray from cover to cover, and yet not know anatomy;" this was the first startling pronouncement that I heard from the eminent anatomist whose assistant I have the honor and good fortune to be. * Had my previous experience been other than it was, I should at once have discounted a statement so sweeping as an overflow from the exuberance of an enthusiastic anatomist. My experience being what it was, however, the mere statement, pregnant with the force and boldness of incontrovertible truth, sufficed without one moment's consideration to flash its full significance on my mind, and to imprint thereupon a biding conviction. For three years previously I had grubbed and garnered all through the pages of Gray, once, twice, three times, but all to no purpose. Those who were then my fellow students, and who were afterwards my fellow graduates,† can bear witness to the fact that to-day, so to speak, after last nights's study of some special region, I would be a perfect cornucopia of anatomical facts, yet within one short week I was sure to be a blank. The next region I studied effaced all trace of the preceding one, and that in its turn gave way to what followed.‡ The anatomy of the leg was unemonically [?] incompatible with that of the arm, while the anatomy of the head and neck could not brook the society of either. (I am speaking now

*[Prof. Wm. Keiller, M. D., Edinburgh, Professor of Anatomy, Medical Department, University of Texas.—ED.]

†[In class of 1891-2, Medical Department, University of Texas.—ED.]

‡Or to quote "Dombey & Son" with regard to little Paul at Dr. Plimmer's, "Didn't know whether twenty Romuluses made one Remus, or whether hic hæc hoc was avoirdupoise or troy weight.—ED.]

of course of anatomy learned from the text-book alone, for owing to special circumstances at the time of my opportunities for dissecting were very limited.) So it was; I could commit to memory and rattle off, but I could not retain; and I have no fear of being confronted with the suggestion that my case might have been in any way exceptional.

The worst of it was that what I did retain availed me nothing; for in presence of the cadaver it all became the sport of a demon of perversity. Everything in the text-book diagrams stood out so clearly and distinctly and kept its place in such an orderly and obliging way that one could not help feeling that fate was against him when the confounded cadaver would maliciously get everything jumbled and indistinct, needlessly mystifying what might just as well have been as simple and well-adjusted as the layers of a dissecting manikin.

How different the result when, with or without the help of the demonstrator, a structure was sought out and laid bare on the cadaver, and there studied with respect to form, action, or relation to other structures! Facts so mastered and appreciated left, not a mere fleeting picture as when studied from the text-book, and diagrams, but rather a clear, well-defined mental impression which, as a rule, was left to stay.

Such was my experience as a student of anatomy, and the lesson it teaches is very plain. To give that lesson adequate expression, I would say, first, that not all the text books that were ever written, and not all the diagrams that were ever traced, even by the wizard fingers of a Keiller, can impart any useful knowledge of anatomy without constant recourse to the *cadaver;* and, secondly, that upon the cadaver, properly dissected, a student may learn the whole of anatomy, even if there were not a single text book, or a single diagram, in the wide, wide world.

The question naturally suggests itself now, is there, then, no place for the text book and diagram in the study of anatomy? Are the universally-admired pages of Gray and of Quain, are the startlingly vivid diagrams of Keiller, to be relegated to the limbo of popular delusions? Not yet awhile, at least not till some enterprising and philanthropic firm brings out that long-felt want, a dissecting manikin, whose anatomy shall excel, in normality and accuracy, that of the most symmetrical and orthodox cadaver in existence, or in pickle. Till that happy day arrives, the text book will be studied both before the dissection and after; but the pleasure and profit derived from the two readings will differ as widely as the pleasure experienced by a hungry man, in poring over the pages of a cookery book, differs from that experienced by him in eating some of the good things for which the book gives standard recipes. As to the diagram, I would say, that if it be a really good one, its use is two-fold: first, for elucidation of the descriptive text, for without it the most lucid text is worthless; second, as the best substitute the dissector can have, when the professor, or demonstrator, is not at his elbow.

To sum up, then, the text book and diagram are useful only so far as they help in the work of dissection, or in refreshing the memory on what has already been properly dissected. Outside of this sphere they may serve to enable one to pass a written examination, but never a bit of real anatomy do they teach.

The text book, the diagram, the cadaver, but the greatest of these three is the cold, wet, "stiff."

Speaking of the "stiff" reminds me of another feature of this question which, though not an active instrumentality in teaching, has yet much to do with success or failure in study. I refer to the condition of the cadaver itself.

Dead men tell no tales, but under certain circumstances they may give rise to the most atrocious smells. What chance, what possibility is there for either teacher or student, to awaken or maintain enthusiasm when, at the very door of the dissecting room, a silent volley of olfactory villainy pours in upon the pigmented area of his Schneiderian membrane? No wonder the study of anatomy is unpopular and neglected in schools where the method of preserving bodies is a lost art, or was never known. No wonder that such graduates of those schools, as are old in prejudice as well as in years, scout the idea of an aseptic dissecting room, or even of one without an offensive odor. Not quite three months ago a somewhat irascible member of the profession almost clawed me for suggesting that such a thing was possible; and to-day, as Demonstrator of Anatomy in this college, I cannot help feeling a little resentment at recalling the fact that students of this school are prohibited from attending a certain class of surgical operations at the John Sealy Hospital if, on the morning of operation, they have previously entered the dissecting room of this building. A straw best shows how the wind blows, and straws of this kind merely serve for eloquent tell-tale evidence of days that, alas! are not yet wholly past, and experiences not yet quite forgotten. You may prate and preach about asepsis and antisepsis, but you will never fully realize what either means till you have examined dissected specimens which, for more than a year, have lain through all the heat, and damp, and fog, of our everchanging climate, on the dissecting tables of the Medical School of the University of Texas.

To end up with somewhat of a digression, I will say that, for my part, if I had to undergo a surgical operation, and other things being equal, I had to choose, for the securing of asepsis, or antisepsis, between the dissecting room of this building and the choicest ward of a hospital, I should prefer to have the operation done up there, where I take my daily luncheon, rather than in the atmosphere of disease germs which, from the very nature of the case, must always pervade a hospital that adequately performs its proper functions. I do not mean to say that such pathogenic germs, as may be conveyed in the atmosphere, can be kept out of a dissecting room any more than out of a hospital, but I do say most emphatically, that if there be anything, short of fire itself, more obnoxious than another to these germs, it must be the properly preserved cadaver, the very thing which is supposed to make the dissecting room essentially septic.

How people can deny this, and then pour their weak-tea solutions of germicides on a suppurating wound, in the hope of destroying pyogenic germs, or on a healthy wound, in the hope of preventing such germs from finding a lodgment, appears to me to be one of those absurd inconsistencies for which man is the most notorious animal. I have been cut with a dissecting knife; I have had various scratches, bruises, and slight lacerations on my hands since I became Demonstrator of Anatomy in this institution,* and I can truly say that, so far from healing by first or last intention, they absolutely healed without any "intention" at all. This was more than I could claim for at least ten years previously.

The moral of it all is that to flee for asepsis from a well-kept dissecting room to the best-kept hospital, is from the frying pan into the fire.

---

*Prof. Keiller's plan of preserving cadavers was given in this JOURNAL in June, 1892, Vol. 7, No. 11, page 425.

